# The cardiovascular risk continuum: Continuing to challenge the primary and secondary prevention dichotomy

**DOI:** 10.1016/j.ajpc.2026.101644

**Published:** 2026-04-23

**Authors:** Harpreet S. Bhatia

**Affiliations:** Division of Cardiovascular Medicine, University of California San Diego Health, 9500 Gilman Drive, MC 7411, La Jolla, CA 92093, USA

The VICTORION-1 PREVENT (V-1P) study is an ongoing, phase 3 clinical trial evaluating inclisiran compared with placebo in individuals at high risk for a first major adverse cardiovascular event (MACE). Inclusion criteria for high risk for a first MACE event include non-obstructive coronary artery disease, calcium score (CAC) ≥100 AU, 10-year estimated ASCVD risk ≥20% or 7.5–<20% with at least 2 risk-enhancing factors. Participants had to also have an LDL-C ≥70 but <190 mg/dL on stable lipid lowering therapy. Notably, participants were excluded if they had CAC of 0 or no atherosclerotic disease on a coronary angiogram or coronary computed tomography angiography scan within 2 years (NCT05739383). This study will help to advance the understanding of the lipid management of individuals in a high-risk primary prevention population.

In the current issue of *AJPC*, the study by Wang, et al (10.1016/j.ajpc.2026.101524), translates the V-1P inclusion criteria to a real-world population, and characterizes the presence of subclinical cardiovascular disease and cardiovascular risk by imaging and blood biomarkers. The study focuses on the 10-year atherosclerotic cardiovascular disease (ASCVD) risk inclusion criteria and uses data from 848 participants in the STANISLAS cohort from France. There are several key findings in this study. First, among the overall cohort, 16% of participants were eligible using the Pooled Cohort Equations (PCE) and 7% were eligible by both the PCE and AHA Predicting Risk of Cardiovascular Disease EVENTs (PREVENT) equations. The PREVENT equations have been associated with lower predicted risk than PCE [1] but improvements in risk prediction [2]. While a lower percentage of participants were eligible based on PREVENT, this amounted to a difference of only 13 participants in the total cohort. Additionally, the 10-year risk thresholds used for V-1P and the current study are based on prior guidelines for the PCE; however, these thresholds have now been lowered for lipid management with PREVENT [3]. Second, there was a higher prevalence of subclinical cardiovascular disease or markers of increased cardiovascular risk among individuals eligible for V-1P compared with those ineligible for V-1P. For example, carotid plaque was present in 30–32% of eligible participants versus 17% of ineligible participants. There were also modest differences in other carotid ultrasound parameters, echocardiographic parameters, and biomarkers associated with cardiovascular risk (such as troponin I, interleukin-6, high-sensitivity c-reactive protein).

There are limitations to the study to consider. The overall cohort was relatively small, and significantly smaller (73 participants) when considering those eligible for V-1P. The study cohort is made up of families from a specific region in France, which may limit the external validity of these findings. Importantly, not all inclusion criteria for V-1P were applied – the study included participants based on 10-year estimated ASCVD risk; however, other inclusion criteria such as CAC or the presence of non-obstructive coronary artery disease, were not evaluated. Thus, this study likely underestimates eligibility for V-1P and is not fully representative of the V-1P population. Related to this, a very important marker of subclinical atherosclerosis, coronary artery calcium scoring, was not evaluated in this cohort. While there are statistically significant differences between the V-1P eligible and ineligible participants in several parameters evaluated, the clinical relevance of many of these is not completely clear. In particular, whether these are tied to important differences in clinical outcomes could not be evaluated as part of this study. Finally, while V-1P eligibility is the focus of the study, the results of V-1P, and whether they drive changes in clinical practice, remain to be seen.

There are several impactful aspects of this study for the field of preventive cardiology. First, the study is an interesting “proof-of-concept” and may be a model for future studies seeking to apply clinical trial eligibility criteria to a real-world population. Indeed, the number of participants eligible for V-1P is striking as this suggests that the inclusion criteria of V-1P will apply to a very large population, making the eventual results of the study impactful, even though the present study likely underestimates the eligible population, as noted above. Second, this study helps illustrate both the positives and negatives of risk scores to guide cardiovascular disease prevention. On the one hand, individuals eligible for the study based on PCE or PREVENT clearly had a higher risk phenotype across several domains. On the other, those who were ineligible for V-1P also had evidence of subclinical disease. For example, 17% of ineligible participants had carotid plaque, demonstrating the potential limitations of a risk factor-based approach to cardiovascular risk assessment.

Most importantly, this study challenges the existing paradigm of primary and secondary prevention. Populations with and without prior ASCVD events are often discussed in guidelines as distinct populations with different recommendations [3]. However, this study adds to the growing body of literature that cardiovascular risk is a continuum rather than a dichotomy. For example, an elegant recent study observed that individuals without a prior event with CAC >300 carry similar future risk to individuals with established ASCVD [4]. Indeed, these findings add to arguments in favor of imaging for subclinical atherosclerosis as a more direct, personalized approach to screening, and highlight the limitations of screening solely based on risk scores, as noted above. In addition, V-1P will help to evaluate whether more aggressive lipid lowering with inclisiran based on imaging for subclinical atherosclerosis will lead to improvement in outcomes. This would align with the results of the VESALIUS-CV trial, which demonstrated significant benefit to evolocumab in another high-risk primary prevention population, the majority of whom had evidence of subclinical atherosclerosis [5]. Future studies, including potential analyses of V-1P and VESALIUS-CV, may consider how outcomes differ based on use of the PCE or PREVENT for eligibility, and based on imaging criteria versus risk-factor based criteria alone.

This study applies clinical trial criteria to a real-world population, demonstrating that the results of V-1P will apply to a large potential population. The study also highlights the limitations of the current approach to cardiovascular disease prevention based on risk factors by demonstrating a high prevalence of subclinical disease in a high-risk primary prevention population, adding to arguments in favor of further personalization, including use of imaging for subclinical atherosclerosis. The results of V-1P are eagerly awaited and, along with the recent publication of VESALIUS-CV, will continue the push away from a primary and secondary prevention paradigm and toward an understanding of a continuum of cardiovascular risk.

## Author agreement statement

We confirm that the manuscript has been read and approved by all named authors and that there are no other persons who satisfied the criteria for authorship but are not listed. We further confirm that the order of authors listed in the manuscript has been approved by all of us. We confirm that we have given due consideration to the protection of intellectual property associated with this work and that there are no impediments to publication, including the timing of publication, with respect to intellectual property. In so doing we confirm that we have followed the regulations of our institutions concerning intellectual property.

We understand that the Corresponding Author is the sole contact for the Editorial process (including Editorial Manager and direct communications with the office). He is responsible for communicating with the other authors about progress, submissions of revisions and final approval of proofs. We confirm that we have provided a current, correct email address which is accessible by the Corresponding Author.

Signed by all authors as follows:

Harpreet S. Bhatia

## CRediT authorship contribution statement

**Harpreet S. Bhatia:** Conceptualization, Writing – original draft.

## Declaration of competing interest

The authors declare the following financial interests/personal relationships which may be considered as potential competing interests:

Harpreet Bhatia reports a relationship with Arrowhead Pharmaceuticals Inc that includes: consulting or advisory. Harpreet Bhatia reports a relationship with Merck Sharp & Dohme UK Ltd that includes: consulting or advisory. Harpreet Bhatia reports a relationship with Novartis Pharmaceuticals Corporation that includes: consulting or advisory. Harpreet Bhatia reports a relationship with NewAmsterdam Pharma Corporation that includes: consulting or advisory. If there are other authors, they declare that they have no known competing financial interests or personal relationships that could have appeared to influence the work reported in this paper.
